# Comparison of the cost effectiveness of pre-service training and in-service training in Ethiopia

**DOI:** 10.1186/2052-3211-7-S1-O18

**Published:** 2014-12-17

**Authors:** Habtamu Berhe, Paul Dowling, Woinshet Nigatu

**Affiliations:** 1 USAID | DELIVER PROJECT, Addis Ababa, Ethiopia

## Background

Ethiopia has implemented the Integrated Pharmaceutical Logistics System (IPLS) since 2009, under the Pharmaceutical Funds and Supplies Agency (PFSA). Although more than 5,000 healthcare workers have been trained on IPLS, staff attrition and expanding service delivery has required ongoing training. To address this, partners provide mainly in-service training (IST); although, recently, pre-service training (PST) has been offered to graduating pharmacy technicians. However, data was not available to compare the cost effectiveness of PST versus IST.

## Method

Graduating pharmacy technicians were given IPLS training in two locations. One year after training, the technicians completed a questionnaire; it included information about their current work place and the relevance of the training to their current roles and responsibilities. Costs to train PST trainees were calculated and compared to costs for IST. An assumption was made that IST and PST training were equally effective provided trainees were hired within one year of graduation.

## Results

Training cost per IST trainee—per diem, transport, meals, trainer costs, and costs from removing trainees from their workplace—was six times that of a PST trainee, which only included trainer time and materials. One year after graduation, approximately 90 percent of PST trainees were working in the healthcare sector. Assuming similar knowledge retention (this was not assessed) PST is almost six times more cost effective. The breakeven point, where IST and PST are equally cost effective, is about 17 percent: if more than 17 percent of PST trainees are hired within one year, PST is more cost effective.

## Discussion

In this instance, assuming knowledge retention levels are similar, PST is a cost-effective solution. PST is cheaper as trainees do not have transport or per diem costs; PST also reduces the time healthcare workers are away from their posts. While relative training costs and recruitment rates will vary from country to country, the data suggests that, in many settings, PST will be more cost effective. However, more research is needed to assess the effectiveness of training: our assumption (which has not been validated) is that training is equally effective if trainees begin work in pharmaceutical logistics within one year of training.

## Lessons learned

Some assessment of comparative training effectiveness should be done to validate the assumption that PST and IST are equally effective, if trainees begin work within one year of training.

